# Klippel-Trenaunay Syndrome Causing Life-Threatening GI Bleeding: A Case Report and Review of the Literature

**DOI:** 10.1155/2013/813653

**Published:** 2013-06-03

**Authors:** Salih Samo, Muhammed Sherid, Husein Husein, Samian Sulaiman, Margaret Yungbluth, John A. Vainder

**Affiliations:** ^1^Department of Internal Medicine, Division of Gastroenterology, Saint Francis Hospital Evanston Program, University of Illinois at Chicago, 355 Ridge Avenue, Evanston, IL 60202, USA; ^2^Department of Internal Medicine, Division of Gastroenterology, CGH Medical Center, 100 East LeFevre Road, Sterling, IL 61081, USA; ^3^Department of Internal Medicine, Division of Gastroenterology, University of Tishreen, Aleppo Street, P.O. Box 2230, Latakia, Syria; ^4^Department of Pathology, Saint Francis Hospital Evanston Program, University of Illinois at Chicago, 355 Ridge Avenue, Evanston, IL 60202, USA

## Abstract

Klippel-Trenaunay syndrome (KTS) is a rare congenital syndrome of vascular malformations and soft tissue and bone hypertrophy. Vascular malformations can affect multiple organ systems. Involvement of the gastrointestinal (GI) tract is uncommon in KTS, but it can be a source of life-threatening bleeding. We report a case of a 32-year-old male with a known diagnosis of KTS who presented with a life-threatening rectal bleeding and was treated with proctosigmoidectomy and massive blood products transfusion. He expired after a long hospitalization. We then review the literature on KTS and management of some of its complications.

## 1. Introduction

Klippel-Trenaunay syndrome (KTS) was first described by two French physicians, Klippel and Trenaunay, in 1900 [1]. The term describes a rare congenital syndrome of venous, lymphatic, and capillary malformations and soft tissue and bone hypertrophy of usually one limb [2] (lower limb is involved more frequently with extension to the trunk). It seems that the right lower limb is affected more frequently than the left lower limb. Patients can be diagnosed with KTS with only one or more of the abovementioned features since patient might not have all the features [3, 4].

Vascular malformations have been reported to affect the gastrointestinal (GI) tract, liver, spleen, and heart and follow a progressive course [2, 5]. Left-sided inferior vena cava (IVC), secondary to persistence of the left and regression of the right supracardinal vein, has been reported [5, 6]. Vascular malformations can also affect genitourinary tract (kidney, bladder, penis, scrotum, vagina, and vulva) and manifest as intrapelvic or retroperitoneal vascular masses [7].

In this paper, we present a case of a 32-year-old male with KTS who presented with a life-threatening rectal bleeding.

## 2. Case Presentation

A 32-year-old male presented with a sudden onset of massive rectal bleeding. He had a history of KTS since childhood. He had multiple admissions for bleeding, presenting mainly as hematuria that started at the age of 11. He also had one episode of self-limited rectal bleeding four years prior to this presentation. His medications were multivitamins with iron supplement.

At the time of admission, physical examination was remarkable for blood pressure of 55/29 mm Hg, HR of 135 per minute, respiratory rate of 22 per minute, and normal temperature. He appeared pale and diaphoretic. Cardiopulmonary examination was unremarkable except for tachycardia and tachypnea. His abdomen was soft and nontender, with active red blood oozing from his rectum. He was noted to have extensive varicose veins over the right leg and scrotum and his right leg was larger than the left one.

Laboratory studies showed Hg 7.1 g/dL (normal 13–17 g/dL), hematocrit 22.9% (normal 38.6–49.2%), MCV 80.6 fl (normal 80–100 fl), MCH 24.9 pg (normal 26–34 pg), platelets 148 k/mL (normal 150–450 k/mL), AST 522 IU/L (normal 0–40 IU/L), ALT 257 IU/L (normal 0–50 IU/L), alkaline phosphatase 39 IU/L (normal 40–129 IU/L), bilirubin 1.7 mg/dL (normal 0-1 mg/dL), and INR 1.7 (normal 0.8–1.2).

**Figure 1:**
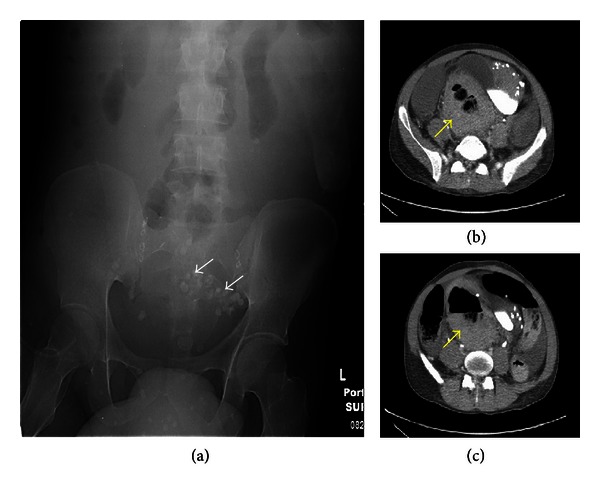
(a) Kidneys, Ureters, and Bladder (KUB) imaging shows numerous venous calcifications consistent with phleboliths from vascular abnormalities in pelvic area (white arrows). (b) and (c) Computed Tomography (CT) scan of the abdomen and pelvis shows markedly thickened colonic (rectal) wall (yellow arrows).

**Figure 2:**
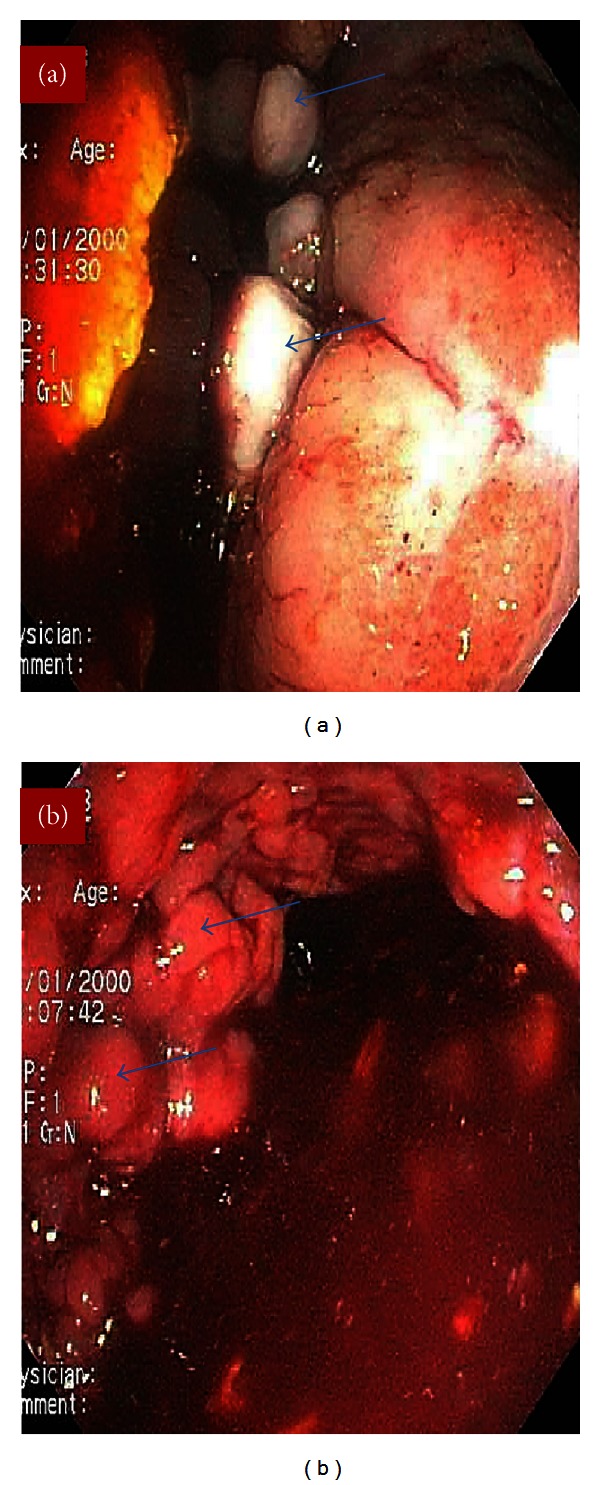
Colonoscopy shows (a) varicose veins (blue arrows) in the rectum and (b) varicose veins (blue arrows) and blood at the hepatic flexure.

Kidneys, ureters, and bladder (KUB) X-ray revealed multiple small rounded calcifications throughout the lower abdomen (Figure 1(a)). Computed tomography (CT) scan of the abdomen and pelvis showed marked wall thickening in the left colon, sigmoid colon, and the rectum (Figures 1(b) and 1(c)). The patient underwent upper endoscopy which was within normal limits. Subsequent colonoscopy showed long, large, and dilated tortuous veins throughout the colon, with large amount of red and clotted blood extending to the ascending colon (Figures 2(a) and 2(b)).

He underwent angiography which showed hypervascularity of one of the superior mesenteric artery branches supplying the right colon. This area was embolized, but the patient continued to bleed and subsequently underwent rectosigmoid resection.

**Figure 3:**
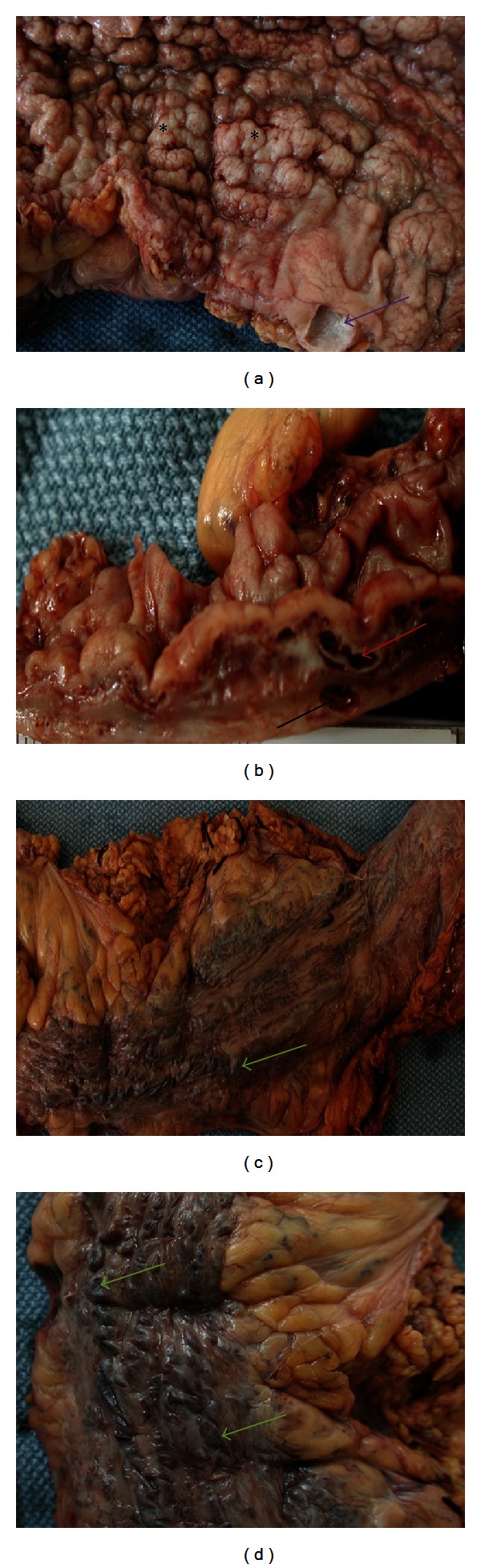
Resected colon (gross) shows (a) colonic mucosa having a nodular surface (asterisks) caused by dilated vascular spaces in the underlying bowel wall. The purple arrow indicates a large lymphatic space in the submucosa which has distorted the overlying mucosa; (b) section through the bowel wall demonstrates dilated blood vessels in the submucosa (red arrow) and muscularis propria (black arrow); (c) and (d) external surface of the resected colon has congested dilated blood vessels consisting of prominent varices on the serosal surface (green arrows).

**Figure 4:**
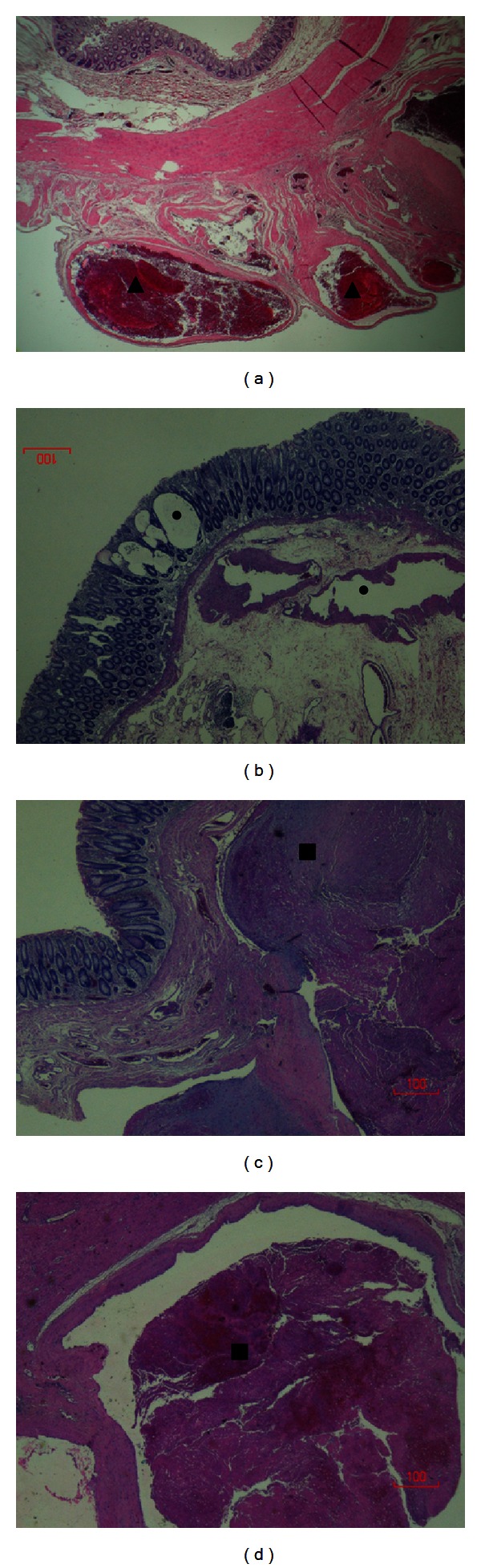
Microscopic examination of the resected colonic segment shows (a) section through the colonic wall which has several dilated blood vessels distorting the serosal surface (black arrowheads) (H&E stain, 10x); (b) several dilated lymphovascular structures in the lamina propria and in the submucosal layers of the colonic wall (black dots) (H&E stain, 10x); (c) and (d) dilated submucosal blood vessels with luminal thrombosis (black squares) (H&E stain, 10x).

Grossly, the resected left colon and rectum showed extensive mucosal varicoses with nodular changes (Figure 3(a)). Almost the entire mucosa was cobblestone in appearance with multiple polypoid areas. The wall was markedly thickened, and the mucosal surface was reddish-pink in color (Figures 3(a) and 3(b)). The serosal surface showed markedly dilated veins with prominent varices (Figures 3(c) and 3(d)). Histological examination of the resected area showed extensive venous malformations and dilated lymphatics with focal thrombosis consistent with KTS (Figure 4). Postoperative course was complicated by hemoperitoneum, worsening coagulopathy, peritonitis, sepsis, and right leg deep venous thrombosis (DVT). He had an inferior vena cava (IVC) filter placed. He was sent to an operative room three times for hemoperitoneum evacuation and wound dehiscence closure. Patient required intubation, mechanical ventilation, and vasopressors multiple times during his hospitalization because of the sepsis and respiratory failure. He required transfusion of a total of 50 units of blood (33 units on the first day of presentation), 18 units of fresh frozen plasma, 26 units of platelets, and 10 units of cryoprecipitate. He expired after 2 months of hospitalization.

## 3. Discussion

Involvement of the GI tract is uncommon in KTS; however, it can be a source of life-threatening bleeding. Only six patients were reported to have rectal bleeding from a study of 588 patients with KTS by Servelle et al. [8]. The source of bleeding from the GI tract is most commonly from the rectum and distal colon [8–12]. GI bleeding from jejunal hemangiomas is rare but has been reported [13, 14]. Esophageal varices secondary to prehepatic portal hypertension from cavernous transformation or hypoplasia of portal vein can be another significant source of GI bleeding [15–17]. Vascular malformations involving the whole GI tract are rare [5].

Bleeding is the most common manifestation of hemangiomas involving the GI tract [18]. Bowel involvement may not be recognized if no overt GI manifestations occur [13]. GI bleeding is usually intermittent and tends to start during the first decade of life [8, 12]. Owing to the intermittent nature of bleeding, it can be misinterpreted as hemorrhoids [19]. Ascites and bowel obstruction may occur from giant hemangioma masses in the retroperitoneal space. However, these manifestations occur less frequently [20].

Since hemangiomas and vascular malformations are managed differently, the initial workup should begin with Doppler ultrasound which can differentiate those two entities depending on high vessel density and high peak arterial Doppler shift, which identify hemangiomas [21, 22]. Phleboliths seen on abdominal radiographs are pathognomonic for hemangiomatosis and venous malformations, and are manifestations of calcified thrombi in KTS [12, 14, 23]. Bowel luminal narrowing with scalloped mucosa can be seen on barium studies. The scalloped appearance of the mucosa is secondary to varicosities or submucosal hemangiomas [12, 24]. CT scan and magnetic resonance imaging (MRI) can assess the intra-abdominal hemangiomas and their extension in the abdomen and pelvis [24, 25] with MRI being used to assess for treatment response and prognosis [26]. Angiogram is fundamental to define the extension of visceral involvement and to help as a surgical guidance preoperatively [11, 13, 14, 27].

Endoscopically, bluish submucosal angiomatous lesions as well as visible mucosal dilated vessels may be seen. Deeper lesions may not be assessed well by endoscopy [18]. The lesions may be mistaken with inflammatory bowel disease if ulceration overlying hemangiomas is present. These lesions should not be biopsied since it may lead to severe bleeding [11, 24]. The whole GI tract should be evaluated endoscopically for the exact localization and extension of the vascular malformations and for effective management of GI hemorrhage [5]. Therapeutic interventional measurements are used when patients do not tolerate endoscopy in case of active bleeding. Angiography can localize the area of bleeding; hemostasis, by arterial embolization or intra-arterial infusion of vasopressin, can be achieved [5, 28].

Patients who are mildly anemic and have good quality of life may be managed with iron supplementation and observation. However, patients with transfusion-dependent anemia or life-threatening hemorrhage need an invasive angiographic arterial embolization or surgical operation [4]. Patients with significant hemorrhage usually require bowel resection to eliminate the source of bleeding [8].

Because of the diffuse hemangiomas and vascular malformations in the bowel, endoscopic therapy usually has a limited role, but it may have a role in postoperative residual or localized lesions. Laser therapy and partial colectomy may be effective in these cases [9, 10].

In KTS, large venous malformations are rare but carry high risk for potential complications secondary to hypercoagulability state and thrombosis. Because physicians are not familiar with these complications, patients may have life-threatening thromboembolic phenomena due to delayed diagnosis [1]. Large venous malformations are associated with low grade coagulopathy (consumptive) [29–31], and patients with larger, more complex vascular malformations have higher risk for thromboembolic disease [1].

Patients of KTS who have thromboembolic disease tend to fail anticoagulation with unfractionated heparin or vitamin K antagonists [1]. Symptoms from intravascular coagulation (localized or disseminated) such as pain should be treated with low-molecular weight heparin and elastic compressions rather than unfractionated heparin or vitamin K antagonists [30]. An IVC filter placement for patients with KTS has high failure rate [32–34]. Failure of the IVC filter is likely due to abnormal venous connection bypassing the filter [35].

In our case, the surgeon was trying to save patient's colon by performing a limited left colectomy, where patient was bleeding profusely. Since patient had vascular malformations throughout his entire colon with ongoing bleeding, we believe that total colectomy should have been performed to avoid more hemorrhagic complications since patient was at high risk for rebleeding from his GI tract. 

In summary, GI bleeding in KTS is uncommon but can be fatal. Therefore, gastroenterologist should be aware of the GI involvement and manifestations in this rare syndrome. Physicians in general should be aware of the nonbleeding complications of KTS since it can increase the morbidity and mortality significantly.
